# Laparoscopic Management of Large Myomas

**DOI:** 10.4103/0974-1216.71611

**Published:** 2009

**Authors:** Rakesh Sinha, Meenakshi Sundaram

**Affiliations:** Consultant endoscopic surgeons, Beams hospitals, Mumbai, India

**Keywords:** Laparoscopic myomectomy, large myomas, fibroids, uterine artery ligation, pregnancy after myomectomy

## Abstract

The objective of this article is to review the different techniques that have been adopted for removal of large myomas laparoscopically. We have also quoted literature about the impact of myomas on Pregnancy and obstetrical outcome and the effect of laparoscopic myomectomy on the same. Technical modifications to remove large myomas have been described along with methods to reduce intraoperative bleeding. This comprehensive review describes all possibilities of laparoscopic myomectomy irrespective of size, site and number.

## INTRODUCTION

Uterine leiomyomas (myomas) are benign smooth muscle tumors arising from the myometrium. Most myomas do not cause clinical symptoms and do not require intervention. Nonetheless, the size and location of a myoma are important determinants of its potential to become symptomatic and cause problems ranging from infertility to life-threatening uterine hemorrhage.

Leiomyomas may develop anywhere in the myometrium and occasionally in the cervix, broad ligament, and ovaries. Most frequently, they develop in the myometrial wall and can lead to uterine distortion (of both the cavity and the overall contour of the uterus), if large and multiple.[1]

Historically, symptomatic myomas have been treated surgically, often by hysterectomy. The surgical management of myomas has advanced significantly with newer, less-invasive forms of therapy. Current options for the management of myomas are numerous and allow for individualized treatment depending on the patient’s desires. Despite these advances, there still are considerable controversies and unanswered questions about optimal surgical or medical management of symptomatic and asymptomatic myomas among physicians. There is no consensus as to when surgical interventions are necessary especially when fertility is desired, what type of therapy is safest or most efficacious, and which treatment option carries the least side effects (either systemic or local such as adhesion formation). This is due partly to the lack of randomized, prospective, well-controlled studies looking at the outcome of treatment for myomas. In the clinical setting, surgical resections of myomas are performed by either open (laparotomy) or endoscopic procedures. Most studies indicate that laparoscopic myomectomy may be an appropriate alternative to abdominal myomectomy in well-selected patients.[2] It is estimated that myomas are clinically apparent in approximately one out of every four to five women of reproductive age[3] and are present in pathologic specimens in up to 80% of surgically removed uteri, independent of preoperative diagnosis. The etiology of leiomyomata is not well understood. The underlying process for the development of myomas requires two distinct events, namely, the transformation of normal myocytes into abnormal myocytes and growth of these abnormal myocytes into clinically detectable tumors. Myomas are usually of monoclonal origin, and growth of the tumor is related to clonal expansion of a single cell. In patients with multiple myomas, each tumor has a different karyotype, suggesting independent formation of each myoma.

In the case of very large myomas, the procedure can be recommended as a routine procedure for patients only after its technical feasibility, complication rates, conversion rate, and long-term outcomes are assessed. The author has reported a case of a large myoma being removed laparoscopically.[4] If the surgeon’s ability to perform the procedure is unlimited, then, size does not matter for performance of a myomectomy laparoscopically.

## SYMPTOMS

**Figure 1:**
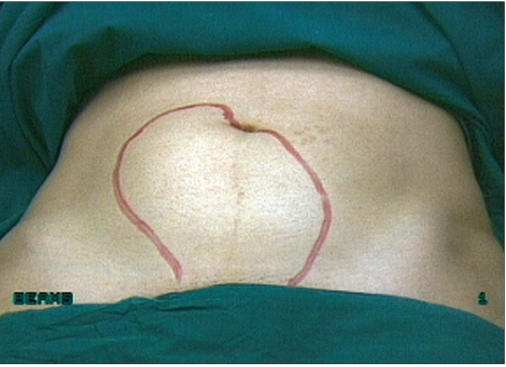
External picture of uterus with multiple fibroids

**Figure 2:**
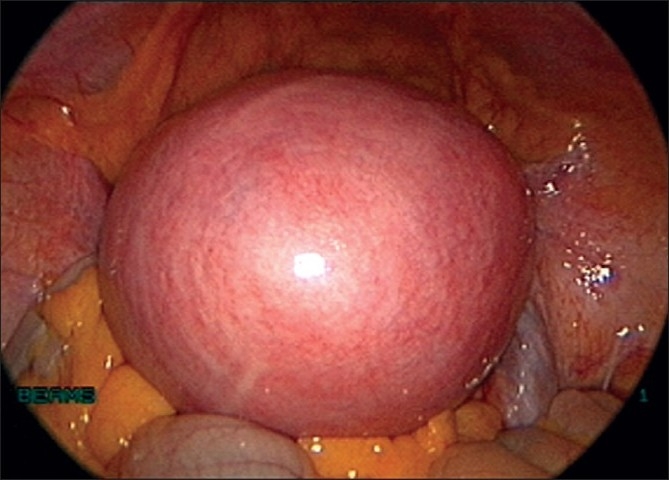
Uterus with large fundal fibroid

**Figure 3:**
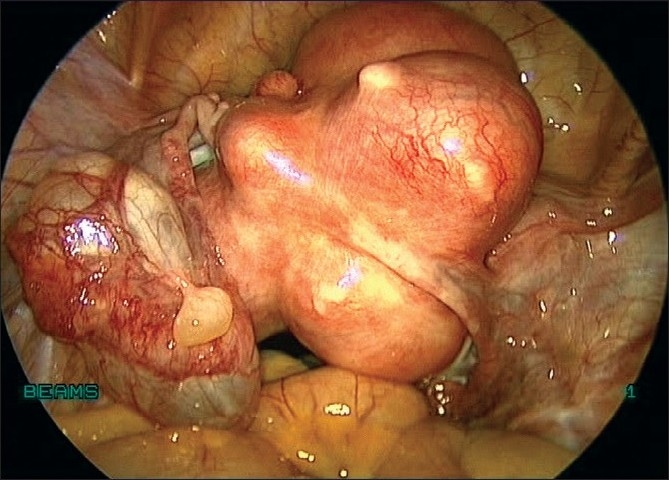
Uterus with multiple fibroids

**Figure 4:**
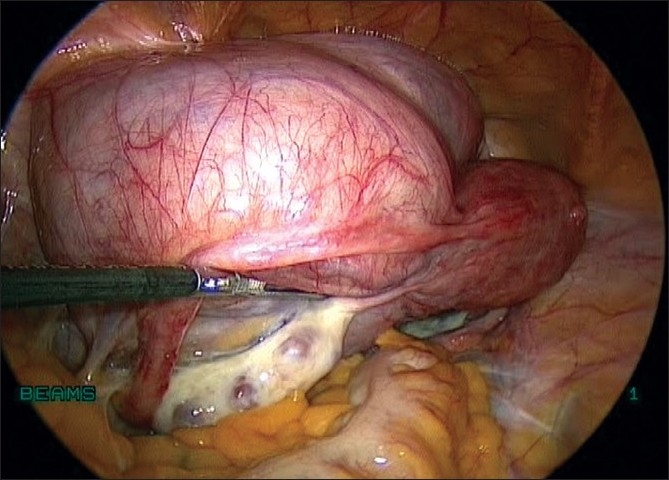
Uterus with Broad ligament myoma

**Figure 5:**
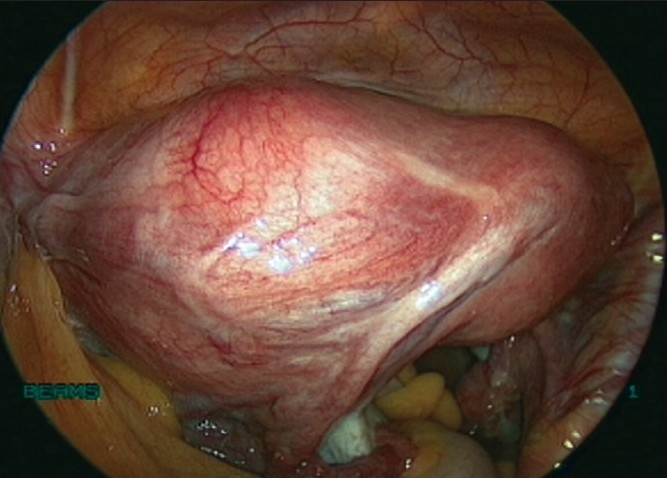
Uterus with lower segment fibroid

The location, number, and size of uterine myomas [Figures 1–5] usually correlate with their clinical significance, with most myomas being small, asymptomatic, and clinically inconsequential. Common problems associated with myomas are pelvic, abdominal, or back discomfort; urinary bladder irritability; abnormal uterine bleeding; bowel dysfunction; infertility; and pregnancy loss and/or complications. These symptoms are usually more pronounced if the myoma is large. Sometimes large subserous myomas may only present as mass abdomen and no other symptoms.

## IMPACT ON INFERTILITY

The speculated mechanisms by which myomas can cause infertility are mechanical interference with implantation, sperm and embryo transport, focal endometrial vascular and endocrine disturbances, endometrial inflammation, and abnormal uterine contractility. The true impact of myomas, despite their location, on pregnancy outcome remains unclear. It can be inferred from studies that a submucosal or cavity-distorting myoma or a large intramural myoma (5–7 cm), whether or not it distorts the cavity or pelvic anatomy, may have a negative impact on reproductive outcome. Submucosal myomas significantly decrease in vitro fertilization (IVF) pregnancy rates. However, only large scale, multicenter, prospective, randomized, controlled studies will be able to decipher how patients should be counseled regarding their options with respect to fertility and myoma management.[5] The majority of the evidence appears to support an impairment of infertility with intramural myoma, particularly in larger leiomyomas.

## IMPACT ON OBSTETRICAL OUTCOME

Women with uterine myomas have a 3.5-fold increase in the incidence of intrauterine growth restriction (6.8%), a fourfold increase in placental abruption (2.8%), a fivefold increase in the incidence of transverse lie or breech presentation (16.9%), a fivefold increase in the cesarean section rate (57.7%), a 70% increase in premature rupture of membranes (9.6%) and are three times more likely to receive transfusion (4.2%).[6]

## MANAGEMENT OF MYOMAS

During the past few years, there have been a number of studies advancing the knowledge about the efficacy and safety of treatments of myomas, including medical and minimally invasive therapies. The mainstay of medical therapy, namely gonadotropin-releasing hormone (GnRH) agonist, reduces myoma size by 30–65% within 3 months of therapy; but this effect is limited, with a rapid return to pretreatment size occurring after therapy along with significant side effects related to the associated hypoestrogenism. The safety and cost effectiveness of this therapy in the long term have not been evaluated, making it unattractive except in perimenopausal women.

Uterine artery embolization (UAE) is an option for women with symptomatic fibroids, who are not candidates for surgery or who do not wish to accept the risks of an operative procedure. The efficacy of UAE for large myomas is questionable. Occlusion of the uterine arteries with a sclerosing substance causes myoma infarction, eventually reducing the size and symptoms of uterine fibroids. UAE is associated with a high clinical success rate and good fibroid volume reduction. Studies on UAE indicate that it is effective in controlling symptoms in 80–94% of women.[7] However, the true incidence of complications is not known because of the small number of patients enrolled in published studies.

## MYOMECTOMY

Criteria for myomectomy for surgical intervention, supported by the American College of Obstetricians and Gynecologists (ACOG) and American society for reproductive medicine (ASRM) are:

- clinically apparent myomas that are a significant concern to the patient even if otherwise asymptomatic;
- myomas causing excessive bleeding and/or anemia;
- myomas causing acute or chronic pain; and
- myomas causing significant urinary problems not due to other abnormalities;
- infertility with distortion of the endometrial cavity or tubal occlusion.

Laparoscopic myomectomy is a controversial procedure, although it is now considered to be feasible.[8] The technique is reputed to be difficult and time consuming and to involve a high risk of conversion to laparotomy. Concerns related to technical difficulty have led to various recommendations based on myoma size, position, and number.[9] It cannot be denied, however, that this procedure has well-known advantages compared with laparotomy. The most common indication is the patient’s desire to avoid hysterectomy and preserve her uterus.[10] Before laparoscopic myomectomy can be recommended as a routine procedure for patients with very large myomas as opposed to laparotomy, its technical feasibility and complications must be assessed.

## OPERATIVE TECHNIQUE

### Preoperative preparation

The patients are kept on a liquid diet for 2 days before the procedure to ensure that bowel loops are empty. Bowel preparation is done. The patients receive prophylaxis against possible thromboembolic episodes with a sequential compression device and subcutaneous injection of low-molecular-weight heparin intraoperatively.

### Procedure

Hysteroscopy is performed in most patients at the outset of the procedure.

### Port placement

Placement of laparoscopic ports is of prime importance as it decides the ease and efficiency of surgery, especially suturing. We perform laparoscopic myomectomy with a 10-mm, 30° foreoblique telescope that provides good visualization of large myomas from various angles. In patients with large myomas, placement of the 10-mm trocar at the usual intraumbilical site could cause the scope to be too close to the fibroid and suture line. The increased magnification would result in a constantly smaller operative field, making precise manipulation of instruments difficult.[11] In such cases, we prefer to place the optical trocar at an appropriate supraumbilical site depending on the size of the uterus and myomas.

**Figure 6:**
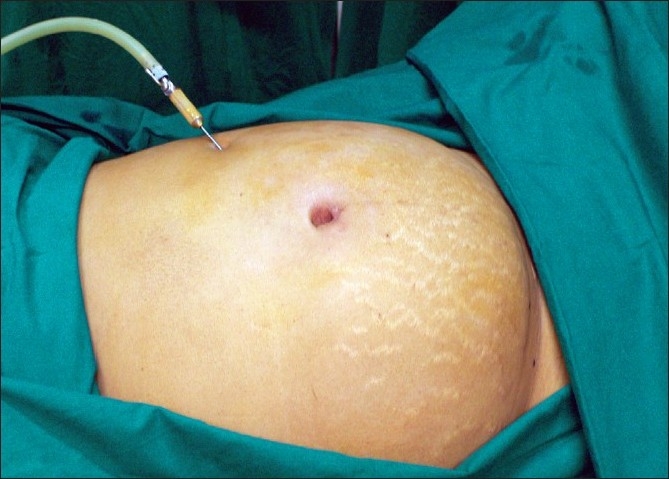
Veress at palmers point

We insert the veress needle at the Palmer’s point to create pneumoperitoneum [Figure 6]. In rare instances where the myoma is extending into the left upper quadrant, veress can be inserted at the corresponding point on the right upper quadrant. A 5-mm trocar is inserted blindly in the left upper quadrant lateral to inferior epigastric vessels and at the level of or above the upper limit of the uterus. If the lesion is very large (extending beyond the umbilicus), we may place both the veress needle and 5-mm port at Palmer’s point.[12]

**Figure 7:**
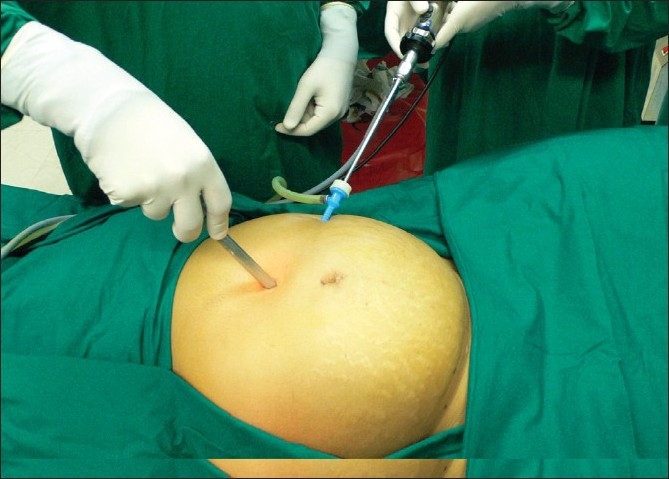
10 mm scope inserted under vision of 5 mm

A 5-mm telescope is inserted through this port and the uterus and myomas are evaluated with respect to size and location. The supraumbilical site for insertion of the 10-mm telescope is chosen depending on the size of the lesion, and the 10-mm trocar is inserted under vision of the 5-mm telescope [Figure 7]. We prefer to place this 10-mm trocar at the supraumbilical location under direct vision to avoid damaging major vessels that are directly beneath the insertion site. The 5-mm port inserted initially can serve as an accessory port for the rest of the procedure. This port has to be placed above or at the upper limit of the uterus so that instruments inserted through it will have unobstructed passage above the fundus of the uterus. An additional 5-mm port is inserted in the right midquadrant of the abdomen lateral to inferior epigastric vessels above the level of the upper limit of the uterus.

We insert another additional port in left lower and lateral aspect, medial to the anterior superior iliac spine. Also one of the left lateral ports is converted to a 15-mm port for the morcellator.

### Enucleation of the myoma

Before myomectomy, all pelvic structures and the abdominal cavity are inspected. The number, site, and location of myomas are noted. If other pathologies are seen, they are usually treated before myomectomy. The course of the ureter, especially in the case of broad ligament myomas, is traced.

**Figure 8:**
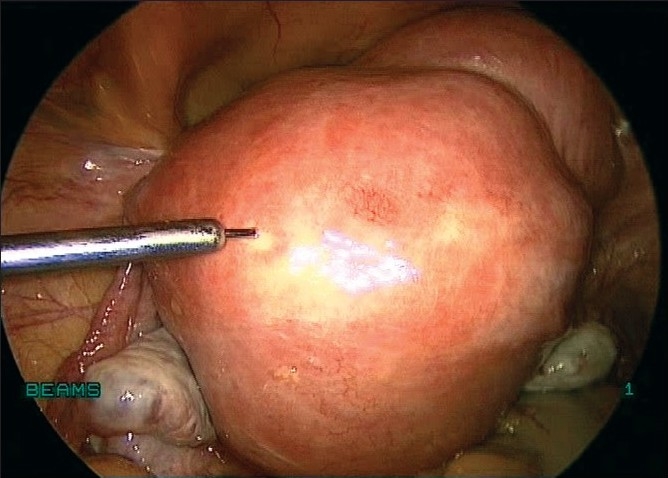
Vasopressin injected

**Figure 9:**
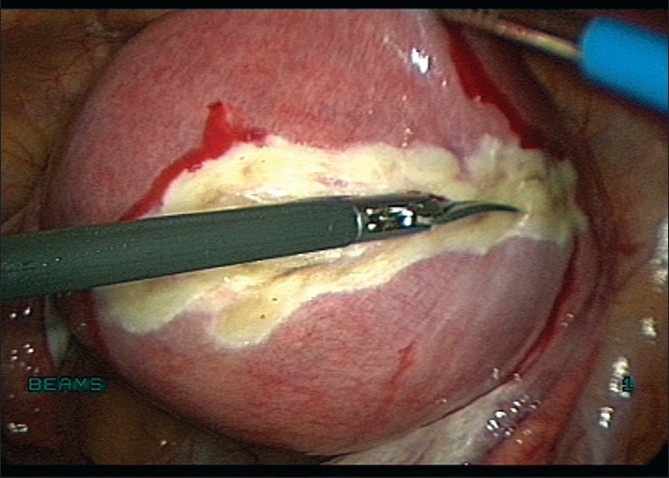
Capsule dissected with bipolar and scissors

**Figure 10:**
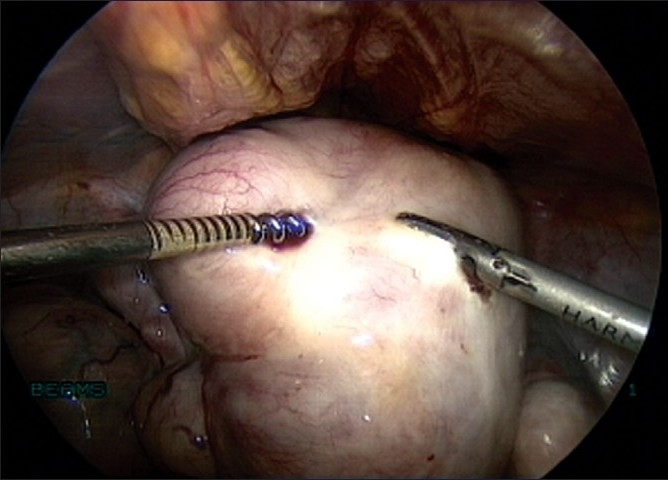
Capsule dissected with Harmonic Ultracision

**Figure 11:**
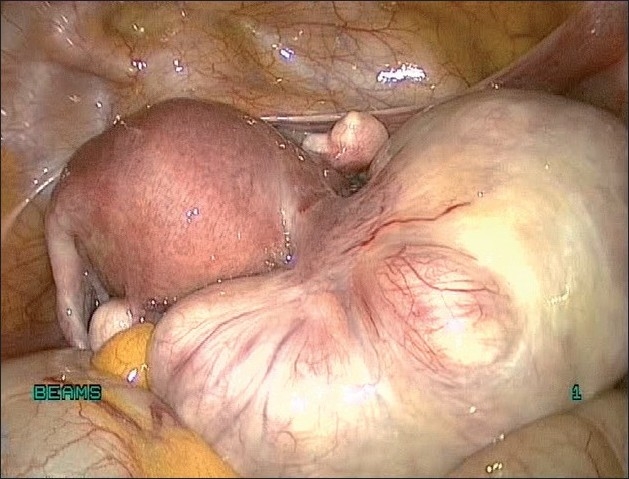
Uterus with Pedunculated fibroid

**Figure 12:**
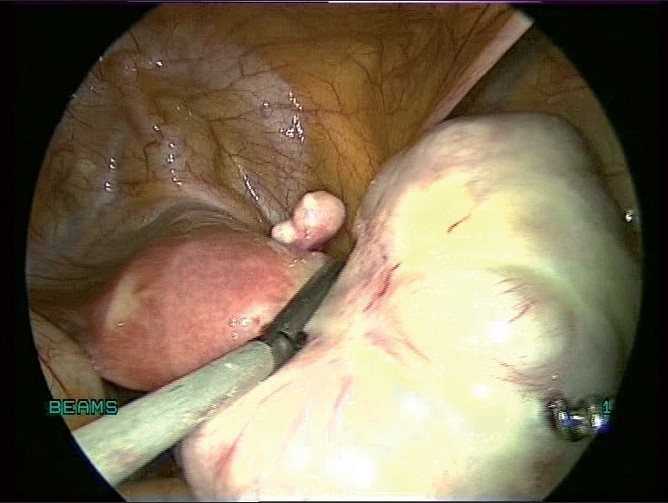
Pedunculated fibroid being clamped

We infiltrate up to 30 ml of vasopressin at a concentration of 10 IU/100 ml of saline solution at several points at the base of the fibroid subcapsularly before the incision [Figure 8]. Conventionally, the incision is made on the most prominent part of the myoma. We prefer to make a horizontal incision on the myoma with bipolar coagulation and laparoscopic scissors or with the harmonic ultracision [Figures 9 and 10], the width of which varies with the size of the lesion. In case of large pedunculated and subserosal myomas, a circumferential incision is made leaving enough capsule for suturing the myoma bed. A pedicle clamp can also be placed in pedunculated myomas and the myoma can be cut off from the base [Figures 11 and 12].

If we separate the myoma from its bed, the excess capsule can be excised together with the myoma. The incision should be large enough to deliver the myoma through it. It is oriented by the ease it would offer in suturing of the uterine wall. In our observation, a horizontal incision offers greatest ease in intracorporeal suturing. It is also associated with less bleeding, as intrauterine vessels run in a horizontal direction.[13] Care should be taken to ensure that the incision does not extend to the cornual end of the fallopian tubes.

In the case of a large anterior wall or fundal myoma, we make a curvilinear incision that does not extend to the cornual ends of the tubes during the process of enucleating the myoma. Bonney’s hood operation can also be done in case of a posterolateral myoma. For intraligamentous leiomyomata, incision of the broad ligament should be large enough to facilitate enucleation of the myoma and allow spontaneous drainage[14] of the blood after surgery. It may be necessary to divide the round ligament to gain access to an intraligamentous myoma.

### Uterine artery ligation

**Figure 13:**
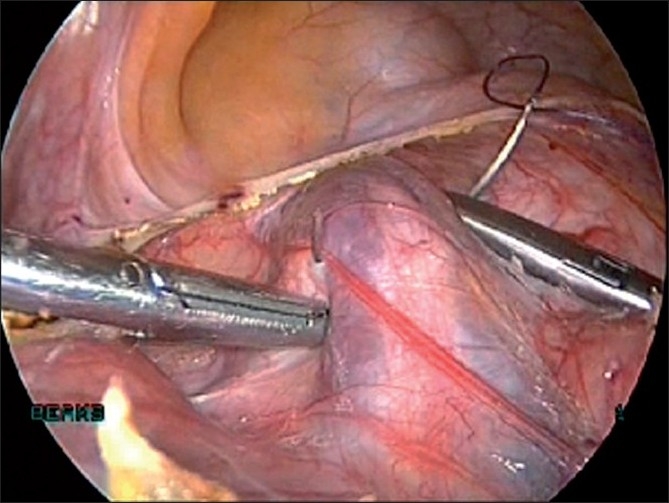
Uterine artery ligation

**Figure 14:**
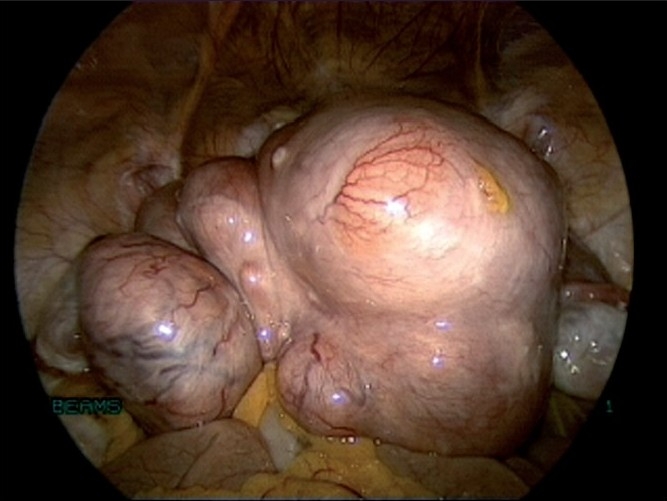
Uterus with fibroids before devascularisation

**Figure 15:**
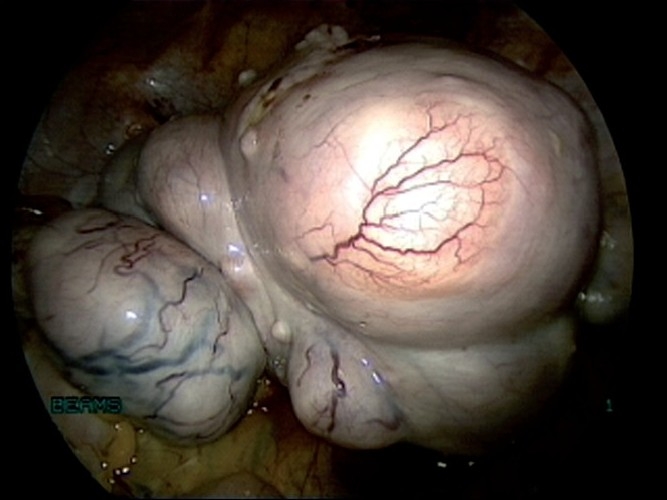
Uterus with fibroids after devascularisation

Laparoscopic ligation of uterine arteries [Figure 13] has been combined with myomectomy with a successful reduction in blood loss.[15] Most cases of large myomas can be devascularized before myomectomy by laparoscopic intracorporeal suturing of uterine arteries. The uterovesical fold of peritoneum is opened and the bladder is pushed down. The uterine vessels are identified on either side and ligated. This devascularizes the myoma and decreases the blood loss during the procedure [Figures 14 and 15].

The vascular supply of the uterus is principally derived from the uterine and ovarian arteries. Because most blood enters the uterus through the uterine arteries, transient uterine ischemia occurs after uterine artery ligation.[16] Bilateral uterine vessel ligation is an efficient method to obliterate the blood flow to the uterus. Leiomyomas derive their blood supply almost totally from the uterine arteries. Devascularization of the myomas by selective uterine artery ligation is the basis for many treatment modalities used for symptomatic myomas, namely, laparoscopic bipolar coagulation of uterine arteries[17] and uterine artery embolization. The author has also reported that ligation of uterine vessels as the first step in total laparoscopic hysterectomy considerably reduces the blood loss during the procedure, especially in cases of large myomas.[18]

**Figure 16:**
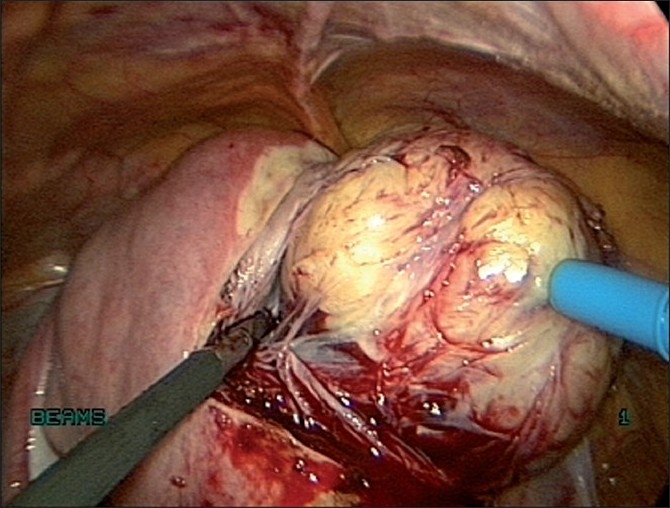
Enucleation of fibroid

**Figure 17:**
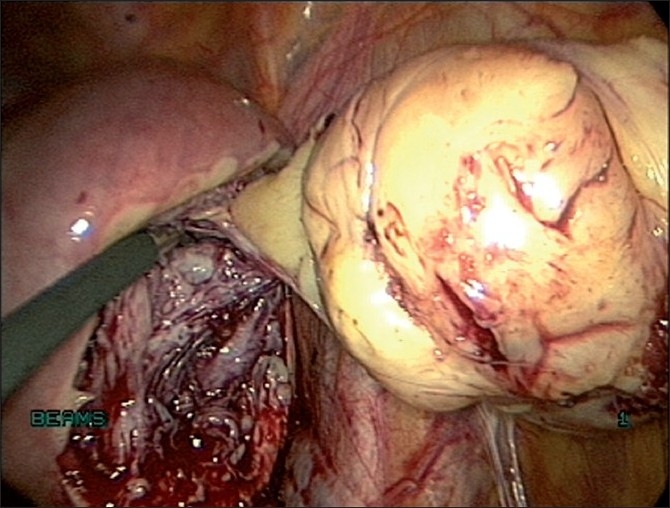
Enucleation of fibroid by traction and counter traction

**Figure 18:**
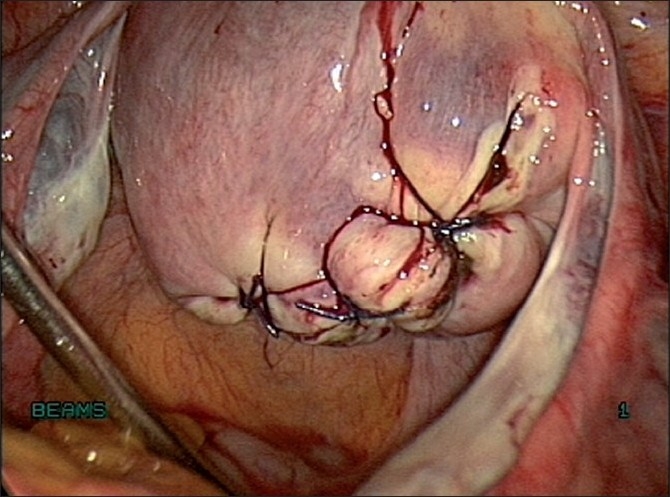
Suturing the myoma capsule

Enucleation is made along the cleavage plane separating the myoma and surrounding myometrium[19] [Figures 16 and 17]. It is facilitated by traction with a 5-mm myoma screw and countertraction on the cervix with a tenaculum held by the assistant. A degenerated myoma may be too friable to allow a firm grip with a myoma screw. Hemostasis is ensured. The myoma bed is obliterated with mattress sutures. The myoma capsule is closed with interrupted intracorporeal sutures with 1-0 polyglyconate in one or two layers depending on the depth of the myoma in the uterine wall [Figure 18]. If the uterine cavity is opened, the endometrium is reposited and the uterine wall is closed excluding the endometrium.

The aim of suture is hemostasis and anatomic apposition. Cheng *et al*,[20] studied the effect of laparoscopic uterine artery occlusion combined with myomectomy for uterine myomas and stated that though hemostasis does not appear to be a problem after artery occlusion, anatomic apposition is the main target of suturing under laparoscopy. Stalks of pedunculated myomas are transected with bipolar coagulation forceps and scissors or with the harmonic ultracision.

### Retrieval of the myoma

**Figure 19:**
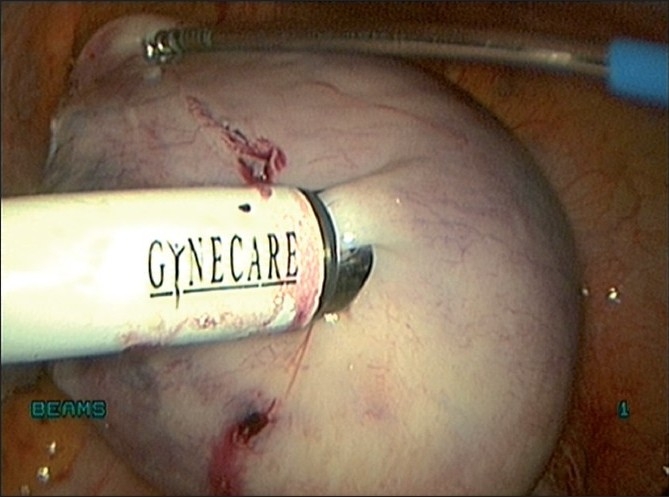
Morcellation

The myoma is retrieved through the 15-mm port by morcellation [Figure 19]. It is important to ensure that all the pieces of the myoma are retrieved. There have been reports of morcellation remnants after myomectomy or hysterectomy, which had developed into myomas and were treated laparoscopically.[21] The 15-mm port is closed with port closure (Reza Granee) needle under vision. The remaining ports are closed with 3-0 polypropylene subcuticular sutures.

Copious lavage of the peritoneal cavity is performed with normal saline solution, approximately 500–1000 ml. The ureters are traced, especially in case of broad ligament myomas.

### Morcellation of the myoma while it is still attached to the uterus

The standard technique of laparoscopic myomectomy requires us to separate the myoma completely from the uterus before morcellation. In symptomatic women who have finished childbearing, the technique used is laparoscopic devascularization of the myomata followed by enucleation of the myomas by direct morcellation.[22]

However, in very large myomas, there is limited space available for the push-pull maneuvers required for the complete enucleation of the myoma. The myoma screw cannot be manipulated with ease, thus increasing the technical difficulty. This is especially the case when dealing with large, softened, degenerated myomas that do not allow adequate grip. The technique of morcellating the myoma while it is still attached to the uterus offers certain advantages that help to overcome these problems.[23] First, it combines two major steps of standard laparoscopic myomectomy: enucleation and morcellation. As the myoma is progressively morcellated and the uterine size reduced, additional space is created for the optimum movement of instruments. Second, the 15-mm claw forceps of the morcellator offers better grip and steady traction even in the case of degenerated myomas. In addition, the electromechanical force exerted by the morcellator causes progressive enucleation of the myoma from its bed even as it is being morcellated, thus completing the process of enucleation. Thus the use of the morcellator to both enucleate the myoma as well as remove it from the abdomen reduces operating time in addition to reducing the technical difficulty of the procedure. A major concern regarding the efficacy of this technique concerns hemostasis. Because the myoma is not completely enucleated before morcellation is begun, one would expect bleeding to be more pronounced because all the feeding vessels have not been coagulated before morcellation. In such cases, prior ligation of uterine vessels reduces the intraoperative blood loss. Large myomas undergo degeneration because of diminishing blood supply and the morcellation of the degenerated areas does not result in excessive bleeding.

One other problem that could arise is in the case of very deep intramural myomas extending up to the endometrium. The traction force exerted by the morcellator could cause the endometrium to be pulled up along with the myoma, and this could result in the inadvertent morcellation of the endometrium along with the myoma, if the surgeon is not alert. The endometrium can, however, easily be distinguished from the myoma, and when the endometrium gets pulled, it can be separated from the myoma.

Small modifications in the technique go a long way in overcoming these problems and decreasing the technical difficulty of the surgery. In our experience,[24] the main procedural details that enabled us to remove even large myomas are the use of a supraumbilical primary port and suitable port geometry, a horizontal incision on the myoma close to the base of the myoma, and the technique of enucleation of the myoma by morcellation while it is still attached to the uterus with or without earlier devascularization.

### Placement of adhesion barrier

**Figure 20:**
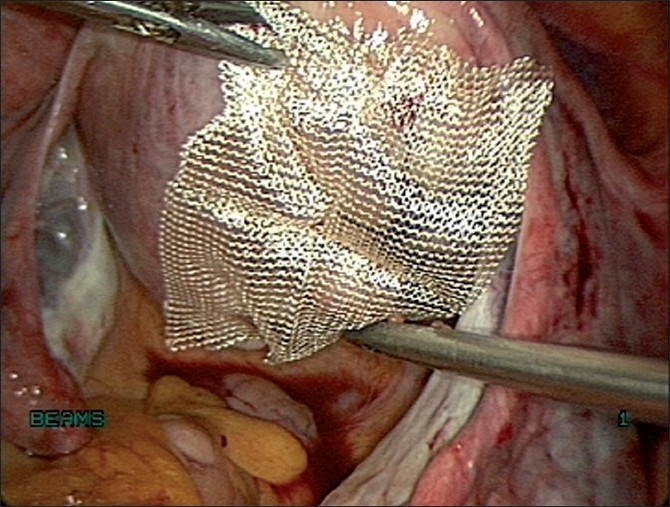
Placement of adhesion barrier

Prospective, randomized controlled studies have evaluated the efficacy of adhesion barriers during laparoscopic myomectomy and found them to be beneficial. The adhesion barrier commonly used is the oxidized regenerated cellulose [Figure 20]. This is placed to cover all incisions and suture material with a 1-cm margin. In a prospective randomized study by Mais *et al*, during second look laparoscopy, 60% of the adhesion barrier group was free of adhesions compared to 12% adhesion free in the control group.[25] Other barriers that possibly reduce adhesions include hyaluronic acid gel and spray gel (synthetic absorbable adhesion barrier).

## FERTILITY AFTER MYOMECTOMY

A recent comprehensive review of the literature (23 studies) on leiomyomas and reproduction reported an overall conception rate of 57% after myomectomy among prospective studies.[26] The conception rate is approximately 53–70% after myomectomy for submucous myomas and 58–65% after myomectomy with intramural or subserosal leiomyomas.

## PREGNANCY AFTER MYOMECTOMY

The pregnancy rate after laparoscopic myomectomy is 54% and comparable to abdominal myomectomy.[27] Studies indicate that laparoscopic myomectomy is a feasible option for infertile women. The best prognosis is found in young women with otherwise unexplained infertility when a myoma distorts the endometrial cavity.

Laparoscopic myomectomy (LM) in infertile patients complicated with uterine myoma is expected to improve the postoperative pregnancy rate as observed with laparotomy.[28] The data are not sufficient to determine whether routine vaginal delivery should be attempted or cesarean section should be advised.

## UTERINE RUPTURE AFTER LAPAROSCOPIC MYOMECTOMY

One of the major concerns about laparoscopic myomectomy in a woman of reproductive age is the risk of uterine rupture during pregnancy or labor due to insufficient closure or healing of a laparoscopic myomectomy incision.

Rupture of the uterus during pregnancy and delivery after laparoscopic myomectomy has been documented as case reports, which describe uterine rupture not only during the process of delivery but also before the onset of labor during the course of pregnancy.[29] It has been reported that uterine rupture occurred in the course of pregnancy or delivery after removal of myomas not only in the deep muscular layer but also of those in the subserosal layer near the surface.

However, when experienced surgeons perform laparoscopic myomectomy, uterine rupture or dehiscence is an infrequent complication. A long-term survey by Dubuisson *et al*, found three cases of spontaneous uterine rupture in 145 pregnancies and only one occurred at the laparoscopic myomectomy site.[30] Based on the clinical trials and case series, it would appear that the risk of uterine rupture during pregnancy is no higher than 1% when the myometrial incision is appropriately repaired.

## CONCLUSION

Laparoscopic myomectomy provides an acceptable, and perhaps a preferable, alternative to abdominal myomectomy for women with symptomatic fibroids who desire uterine preservation and who have infertility primarily related to fibroids. Laparoscopic myomectomy clearly provides a more rapid recovery, less blood loss and fewer adhesions compared to an open approach. Pregnancy rates are comparable to those expected with abdominal myomectomy and the risk of uterine rupture during pregnancy is less than 1% if the uterus is closed appropriately. Meticulous repair of the myometrium using microsurgical principles is essential for women considering pregnancy to minimize the risk of uterine rupture. Adhesion barriers appear to limit postoperative adhesions. A critical issue is the skill necessary for the operating surgeon. Literature reports conversion rates varying from zero to 28.7%, with most conversions largely because of intraoperative bleeding. Each surgeon has to determine selection criteria based on personal proficiency, especially intracorporeal suturing. However, we believe that with requisite skills and good support, the size and location of myomas need not be limiting factors for the procedure.
